# Integration of X-Ray CT, Sensor Fusion, and Machine Learning for Advanced Modeling of Preharvest Apple Growth Dynamics

**DOI:** 10.3390/s26020623

**Published:** 2026-01-16

**Authors:** Weiqun Wang, Dario Mengoli, Shangpeng Sun, Luigi Manfrini

**Affiliations:** 1 Department of Agriculture and Food Science, University of Bologna, 40127 Bologna, Italy; wfw5324@psu.edu (W.W.); luigi.manfrini@unibo.it (L.M.); 2 Department of Electrical, Electronic and Information Engineering, University of Bologna, 40136 Bologna, Italy; 3 Department of Bioresource Engineering, McGill University, Montreal, QC H3A 0G4, Canada

**Keywords:** X-ray computed tomography, apple growth evaluation, machine learning, precision orchard management

## Abstract

Understanding the complex interplay between environmental factors and fruit quality development requires sophisticated analytical approaches linking cellular architecture to environmental conditions. This study introduces a novel application of dual-resolution X-ray computed tomography (CT) for the non-destructive characterization of apple internal tissue architecture in relation to fruit growth, thereby advancing beyond traditional methods that are primarily focused on postharvest analysis. By extracting detailed three-dimensional structural parameters, we reveal tissue porosity and heterogeneity influenced by crop load, maturity timing and canopy position, offering insights into internal quality attributes. Employing correlation analysis, Principal Component Analysis, Canonical Correlation Analysis, and Structural Equation Modeling, we identify temperature as the primary environmental driver, particularly during early developmental stages (45 Days After Full Bloom, DAFB), and uncover nonlinear, hierarchical effects of preharvest environmental factors such as vapor pressure deficit, relative humidity, and light on quality traits. Machine learning models (Multiple Linear Regression, Random Forest, XGBoost) achieve high predictive accuracy (R^2^ > 0.99 for Multiple Linear Regression), with temperature as the key predictor. These baseline results represent findings from a single growing season and require validation across multiple seasons and cultivars before operational application. Temporal analysis highlights the importance of early-stage environmental conditions. Integrating structural and environmental data through innovative visualization tools, such as anatomy-based radar charts, facilitates comprehensive interpretation of complex interactions. This multidisciplinary framework enhances predictive precision and provides a baseline methodology to support precision orchard management under typical agricultural variability.

## 1. Introduction

Apple (*Malus domestica* Borkh.) production requires precise quality assessment and growth monitoring to meet market demands [1,2]. As one of the most economically important fruit crops worldwide, maintaining consistent fruit quality remains a critical challenge in commercial apple production [3,4]. Traditional evaluation methods primarily focus on physical and external attributes through visible observations and measurements [5,6], yet these approaches inadequately assess internal quality parameters [7,8]. Recent advances in precision phenotyping have introduced non-destructive monitoring techniques on spatial and temporal scales [9], with particular emphasis on understanding how crop load and environmental factors influence fruit development on tree [10,11]. Studies have demonstrated that fruit quality is significantly affected by management practices during critical growth periods [10,12,13], especially in the pre-harvest phase, where resource allocation substantially impacts final fruit characteristics [14,15]. Growth mechanisms in apple fruit at different phenological stages and the relationship between key physiological parameters and cellular-level changes can lead to higher quality and a more profitable and sustainable production [16].

X-ray computed tomography (CT) has revolutionized post-harvest fruit analysis by enabling non-destructive internal quality assessment [17,18]. While initial applications focused on detecting internal disorders [8,19], recent studies have demonstrated CT’s capability to analyze a two-dimensional image and three-dimensional cellular architecture and void space distribution [7,20,21]. However, most CT applications have been limited to post-harvest analysis, leaving a significant research gap in their application for monitoring fruit development during growth stages [22,23]. The potential of CT for precise structural analysis during fruit development represents an unexplored frontier in agricultural science [7,24].

The integration of machine learning with environmental monitoring has emerged as a powerful tool for precision orchard management [5,19]. Recent studies have demonstrated the effectiveness of artificial intelligence in predicting fruit quality parameters and optimizing growing conditions using deep neural network models such as Faster R–CNN and Mask R–CNN or Random Forest Trees [25,26]. For instance, González-Pérez [3] successfully employed advanced regression algorithms to predict apple fruit development using environmental data, establishing significant correlations between growing conditions and fruit characteristics. Similarly, Nunes [17,27] integrated multiple environmental variables to model apple quality development, establishing quantitative relationships between growing conditions and final fruit characteristics. These applications demonstrate the potential of machine learning approaches in capturing complex developmental patterns and providing data-driven insights for orchard management decisions [11,24].

Despite significant advances in fruit quality assessment techniques, quantifying complex relationships between environmental conditions and internal fruit development remains a fundamental challenge in pomology research [10,12]. Traditional methodologies lack the precision to correlate cellular-level modifications with environmental parameters [7], while existing imaging techniques cannot effectively capture dynamic structural changes during fruit development [6,17]. The integration of high-resolution X-ray computed tomography with advanced machine learning approaches offers unprecedented opportunities to address these limitations [19,28]. Therefore, this study aims to:

(1) Establish a high-precision X-ray CT framework for quantifying apple fruit cellular architecture dynamics, enabling non-destructive assessment of treatment-dependent structural modifications.

(2) Develop an innovative anatomy-based quality assessment methodology that integrates cellular parameters with environmental variables through radar chart visualization, providing comprehensive insights into fruit quality development.

(3) Implement sophisticated machine learning algorithms to elucidate stage-specific environmental influences on fruit development, establishing a predictive framework for precision orchard management.

This manuscript is structured to systematically present our innovative methodology and findings. Section 2 delineates the comprehensive experimental framework, which includes high-precision X-ray CT protocols, novel anatomy-based quality assessment methodologies, and advanced machine learning algorithms. Section 3 presents quantitative analyses of cellular architecture dynamics, environmental factor interactions, and fruit quality development, supported by multi-dimensional statistical evidence. Section 4 synthesizes these findings through critical discussion, establishing the hierarchical influence of environmental factors on fruit development, and validating our integrated analytical approach. Finally, Section 5 contextualizes the significance of this research in advancing precision orchard management and adapting fruit production systems to climate change scenarios.

## 2. Materials and Methods

**Figure 1:**
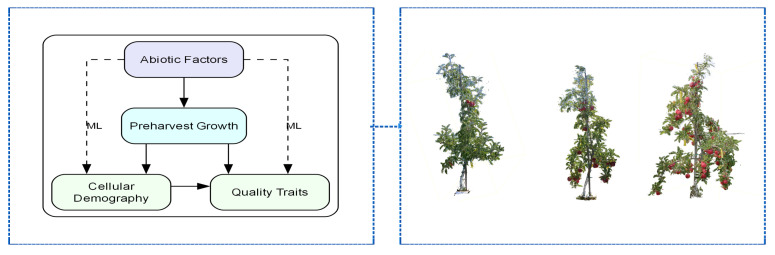
(**left**) Research Framework; (**right**) Low-Standard-High Crop load Apple Trees.

This investigation examines the intricate interaction between preharvest environmental factors and the development of apple fruit through an innovative dual path analytical framework that encompasses both cellular demography and preharvest growth dynamics (Figure 1left). By applying statistical methods to evaluate abiotic-apple quality dynamic relationships and utilizing advanced machine learning algorithms to predict fruit quality development [5,8,17,19].

### 2.1. Experimental Design and Treatments

The work was conducted on eight-year-old ‘Gala’ apple trees (*Malus domestica*) in a 2.6-hectare commercial orchard at McGill University Macdonald Campus, Canada (45.4099° N, 73.9337° W, 35 m). The site has a mean annual temperature of 6.8 °C, 2051 daylight hours, and 1050 mm annual precipitation. The trees grafted on the M106 rootstock were planted at a density of 2200 trees/ha (0.8 m × 3 m spacing) in loamy-silty sand soil with a 4° northward slope.

The experimental protocol included three primary treatment factors. Crop load management (Figure 1right) involved manual thinning in early July when fruit diameter reached 20–30 mm. Crop load treatments consisted of high (92 fruits/tree, trunk cross-sectional area: 18.10 cm^2^), standard (46 fruits/tree, 15.90 cm^2^), and low (23 fruits/tree, 16.61 cm^2^) density levels. The effects of canopy position were evaluated at three locations: upper canopy (P1), eastern lower canopy (P2), and western lower canopy (P3), with triplicate sampling at each. Temporal maturity was assessed at three harvest intervals: Early season (T1: 30 August), mid-season (T2: 16 September) and late season (T3: 30 September 2024), each with three replicates.

Fruit harvesting was performed during pre-sunrise conditions (before 10 AM) between 100–134 days after full bloom (DAFB), spanning from 30 August–30 Septembe 2024. Each harvest event yielded nine apples, with three replicates of 27 samples collected and maintained under controlled conditions (4 °C, 40% relative humidity) in McGill University’s Department of Bio-resource Engineering pending analysis.

### 2.2. Preharvest Environmental Factors Monitoring

Environmental monitoring was carried out at the “STE-ANNE-DE-BELLEVUE 1” meteorological station in Quebec (45°25′38″ N, 73°55′45″ W) Canada, positioned approximately 3 km from the experimental orchard. The comprehensive data acquisition period extended from 20 May to 30 September 2024, corresponding to 0–134 days after full bloom (DAFB). The monitoring protocol encompassed a suite of atmospheric parameters, including air temperature fluctuations, precipitation patterns, accumulated growing degree days, relative humidity dynamics, and wind characteristics. Supplementary environmental metrics were derived through computational analysis, incorporating crop-intercepted radiation, reference evapotranspiration (ET_o_), and vapor pressure deficit (VPD). VPD calculations were performed using the established Tetens formula [5,29]. The soil moisture dynamics was continuously monitored through a network of dual sensor systems (Arduino, Monza, Italy) strategically positioned 20 cm from each tree trunk at a depth of 15 cm. These sensors captured high-resolution temporal data at 30-min intervals throughout the preharvest period [15] as these parameters have demonstrated linear relationships with fruit development during the preharvest stage [3,11].

### 2.3. Fruit Quality Assessment

#### 2.3.1. Maturity Test

Fruit maturity parameters were quantitatively evaluated using precision-calibrated instrumentation: Morphological measurements were obtained using a digital caliper (Mitutoyo Corp., 20-1, Sakado 1-Chome, Takatsu-ku, Kawasaki-shi, Kanagawa 213-8533, Japan), while mechanical properties were assessed through flesh firmness determination using an Instron ElectroPuls equipped with a 7.5 mm probe operating at 50 N [6,29]. Surface colorimetric analysis was performed using a Minolta CR-300 tristimulus colorimeter, and soluble solids content was determined using an R2 Mini refractometer (Reichert, Depew, NY, USA). Fresh weight and seed morphological characteristics were quantified using a high precision electronic scale (Model-121, Acculab, San Diego, CA, USA, ±0.01 g). Principal Component Analysis (PCA) was employed to analyze the collected quality parameters [5,28,30], resulting in the identification of three distinct quality groups [9,31]. This multivariate approach enabled the comprehensive visualization of quality parameter relationships and their relative contributions to overall fruit quality variation. The PCA-based quality assessment framework provided an objective method for fruit quality classification, revealing natural clustering patterns between the measured parameters [24,32].

#### 2.3.2. Anatomy-Based Radar Chart Analysis

This work developed a novel radar chart methodology to visualize multidimensional quality parameters based on PCA results [28,30]. The quality parameters were integrated into a comprehensive index:

(1) $$Q=\sum_{i=1}^{n}\left(w_{i}\times z_{i}\right)$$

where *Q* represents the overall quality index, $w_{i}$ is the weight assigned to each parameter, and $z_{i}$ is the standardized value of the *i*th quality parameter. The weights ($w_{i}$) were determined based on the PCA loading patterns, with higher weights assigned to variables demonstrating stronger loadings on the principal components. This weighting scheme ensures that the quality index *Q* reflects the natural variance structure inherent in the apple quality dataset, providing an objective and data-driven representation of relative parameter importance. The radar chart visualization was structured according to the natural groupings identified through PCA, enabling an intuitive representation of quality parameter interactions across different treatments and developmental stages [31]. The clustering patterns revealed by PCA provided the foundational structure for the radar chart organization, facilitating visualization of complex quality relationships [5,32].

### 2.4. X-Ray Computed Tomography

**Figure 2:**
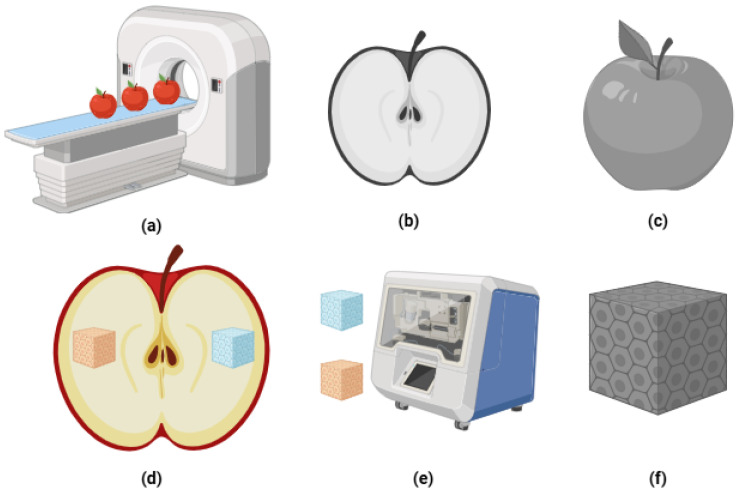
Overview of Apple X-ray CT Imaging: (**a**) Low-resolution scans (339 µm) of three apples; (**b**) corresponding grey value DICOM images; (**c**) 3D reconstruction; (**d**) 1 cm^3^ samples from sunlit and shaded sides; (**e**) high-resolution (6.6 µm) imaging of two samples per scan; (**f**) analysis of cellular and pore structures.

Apple tissue architecture was characterized through a dual-resolution X-ray CT approach (Figure 2), integrating low-resolution porosity distribution mapping with high-resolution cellular characterization [7,8,17,19].

#### 2.4.1. Linear Regression Between Grey Value and Apple Porosity

The Grey value analysis was conducted using int16 scale (−32,768 to +32,767), which demonstrates a strong correlation with apple tissue porosity [7,21]. This established apple juice as the reference point 0% and external air as 100% porosity (R^2^ = 0.9571) using int8 (−128 to +127) [7,17]. This study adapted this approach, utilizing ‘Gala’ apple (*Malus domestica*) juice as a reference point with 0% porosity. The linear regression model was constructed using these two reference points, with subsequent validation performed using random sampling of 10 pixels from the DICOM scan data [28]. This approach follows established protocols from prior X-ray CT studies [7,8], which have demonstrated 10-pixel validation as sufficient for rapid grey value-porosity correlation calibration across fruit types.

#### 2.4.2. Low-Resolution Scanning of the Intact Apple for Porosity Analysis

Low-resolution analyses were executed using a Canon Aquilion Prime SP system (Canon, Tokyo, Japan), enabling simultaneous scanning of three apples. The scanning protocol employed operational parameters of 80 kV and 50 mA, achieving a voxel resolution of (339 μm). A total of 27 intact apples underwent scanning across 9 sessions (three apples per scan). Each 10–13 s scanning sequence generated approximately 1200 projection images (512 × 512, int16 scale) in DICOM format, maintaining 0.5 mm inter-scan thickness. Three-dimensional volumetric reconstructions were generated through Python (3.9.20) for comprehensive porosity distribution analysis [7,22].

#### 2.4.3. High-Resolution Scanning of Cube for Cellular & Pore Architecture

Nine apples were selected for microscale analysis based on the experimental design parameters (two samples per apple, totaling 18 samples). High resolution characterization was performed on 1 cm^3^ tissue samples extracted from sunny and shady apple regions using a SKYSCAN 1174 system (Bruker, Kontich, Belgium). Optimal scanning parameters were established at 50 kV and 604 μA, achieving (6.6 μm) resolution [7,33]. Due to equipment constraints, the paired samples were scanned simultaneously in vertical alignment, with 9 scanning sessions conducted over 9 h. The scanning protocol incorporated 0.5° rotation steps over 360° with 1600 ms exposure and three-frame averaging. Cellular characterization focused on a 0.5 × 0.5 × 0.5 mm region of interest [8,34].

#### 2.4.4. Image Processing

The image analysis workflow for low-resolution DICOM files encompassed individual apple segmentation followed by Otsu’s thresholding for cell-pore differentiation [5,35]. Porosity was determined via linear regression calibrated with apple juice (Int16 value: 49, 0% porosity) and air (Int16 value: −1007, 100% porosity), within the Int16 range of −32,768 to +32,767 [7,36]. High resolution scan processing incorporated 3D ROI definition, contrast enhancement, and blob detection methodologies, supplemented by Python-based (3.9.20) boundary refinement. Methodological validation was achieved through comparative analysis, maintaining consistent voxel parameters on resolution scales [22,37].

### 2.5. Statistical Analysis

Treatment effects were evaluated using one-way analysis of variance (ANOVA) with Tukey’s post hoc test for multiple comparisons [11]. Statistical significance was established at p<0.05. For anatomy-based quality assessment, we developed a novel radar chart methodology incorporating standardized quality parameters [28,30]. The standardization process employed the Z-score transformation:

(2) $$Z=\frac{X-\mu }{\sigma }$$

where *X* represents the original value, μ is the population mean and σ is the standard deviation. This standardization enabled direct comparison of parameters across different scales and units [31].

### 2.6. Correlation Analysis and Predictive Modeling

#### 2.6.1. Correlation Analysis

The complex relationships between environmental factors and apple quality parameters were investigated through multiple analytical approaches encompassing 14 environmental parameters, including temperature regimes, precipitation patterns, humidity dynamics, and solar radiation metrics. Generalized Additive Models (GAM) were employed to capture non-linear relationships between environmental variables and quality parameters, enabling flexible model specification without assuming linear relationships [3]. Spearman correlation analysis was conducted to assess the monotonic relationships between the variables, with significance determined at p<0.05. To further unravel the complex interactions, we implemented multidimensional analyses through a comprehensive framework. Canonical Correlation Analysis (CCA), an advanced multivariate technique that identifies relationships between paired variable sets [3], Partial Least Squares (PLS) regression to handle multicollinearity among predictors [1,11], and Structural Equation Modeling (SEM) to establish causal pathways between environmental factors and quality parameters [5,10,27].

#### 2.6.2. Predictive Modeling Framework

The predictive modeling framework incorporated three machine learning algorithms: Multiple Linear Regression (MLR) [5,30], deep neural networks (Faster R–CNN and Mask R–CNN) [25], Random Forest Regression [26], and XGBoost [19]. Model performance was rigorously evaluated using three complementary metrics: coefficient of determination (R^2^), Root Mean Square Error (RMSE), and Mean Absolute Error (MAE). Feature importance analysis was conducted to identify key environmental determinants at different developmental stages, while temporal pattern analysis was performed by evaluating model performance across distinct developmental periods (45–90–134 DAFB), enabling the identification of stage-specific environmental influences on fruit quality development [11,26,38].

## 3. Results

### 3.1. X-Ray CT Analysis of Apple Tissue Architecture

#### 3.1.1. Distribution and Determinants of Fruit Porosity

**Figure 3:**
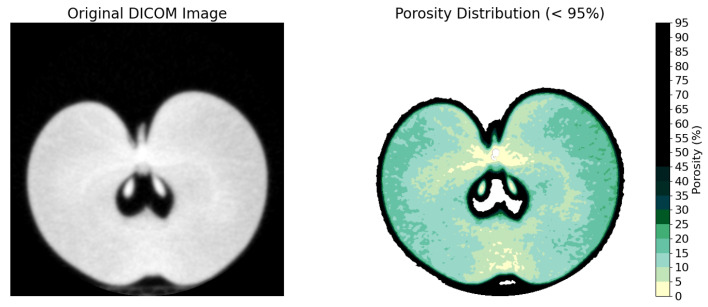
Two-dimensional porosity distribution example.

**Figure 4:**
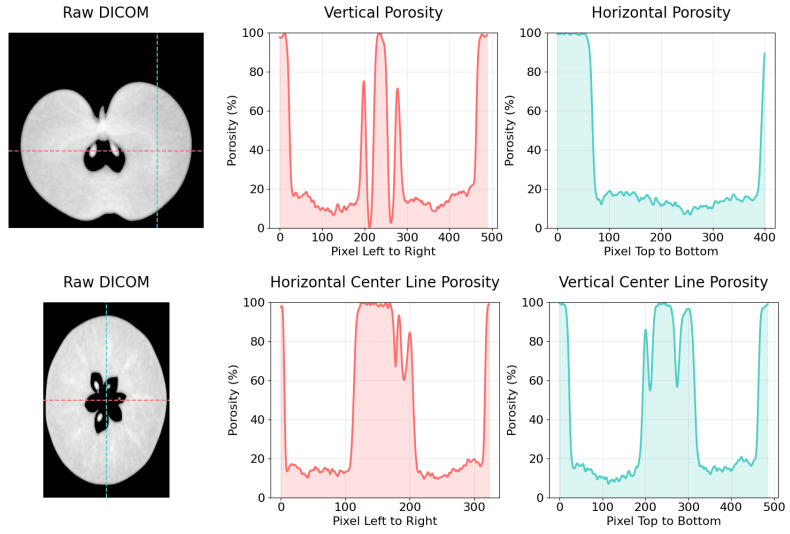
Porosity analysis of both cross and longitudinal section of apples across highlighted lines.

**Figure 5:**
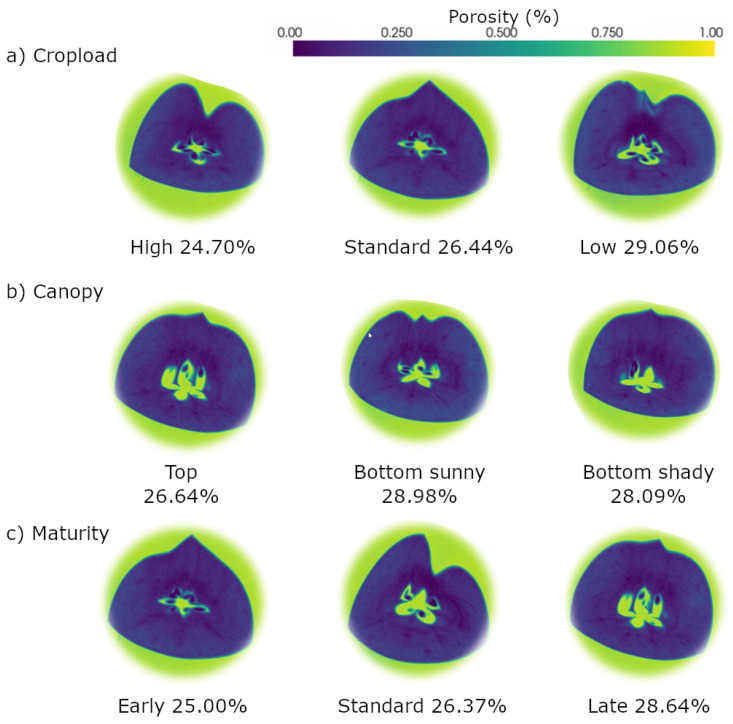
3D section porosity comparison across different samples in terms of (**a**) cropload, (**b**) canopy position and (**c**) maturity stage.

Spatial distribution analysis demonstrated heterogeneous porosity patterns within fruit tissue cross-sections (Figure 3), with porosity values exhibiting a hierarchical progression from seeds (0–10%), core tissue (5–15%), cortex (15–30%), to cavity spaces (100%), while intact regions maintained an average porosity of 20–30%. Both cross-sectional and longitudinal analyses (Figure 4) elucidated elevated porosity concentrations in the core region and subepidermal zones, contrasting with diminished values in cortical tissue, thereby providing comprehensive two-dimensional porosity gradients from superior to inferior positions and across the fruit diameter. The three-dimensional positional analysis (Figure 5) revealed distinct average porosity variations across three experimental parameters: crop load, canopy position, and maturity stage. The crop load demonstrated an inverse relationship with porosity, where high crop load yielded the lowest porosity (24.70%), followed by standard (26.44%) and low crop loads (29.06%). The canopy position analysis indicated minimal porosity in top-positioned fruits (26.64%), with elevated values in sun-exposed (28.98%) and shaded positions (28.09%). Furthermore, fruit maturity exhibited a progressive increase in porosity from early (25.00%) through middle (26.37%) to late harvest stages (28.64%), demonstrating the temporal evolution of tissue structure.

#### 3.1.2. High Resolution Microstructural Analysis

**Figure 6:**
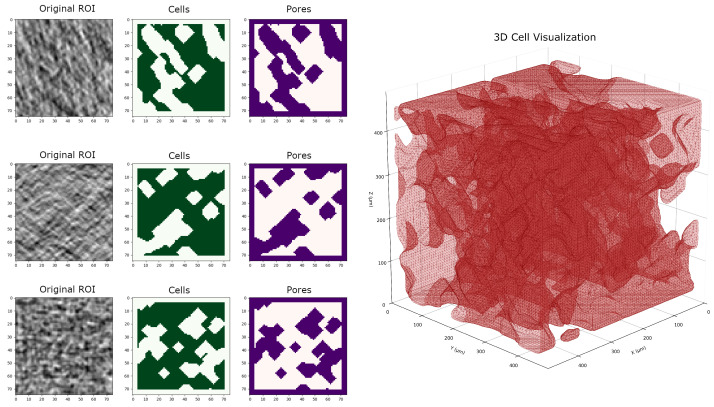
High-Resolution X-ray CT cell analysis showing the 1cm cube ROI within the apple.

High-resolution cross-marker analysis facilitated the differentiation between cellular structures (bright regions) and intercellular spaces (dark regions) within apple tissue. The three-dimensional image segmentation (Figure 6) quantified the critical architectural parameters, revealing a mean cell diameter of 69.0 ± 1.2 μm and a cell density of 1.6 × 10^3^ cells/mm^3^. Further statistical analysis of position-dependent variations indicated that sun-exposed regions exhibited 1.71% higher porosity compared to shaded areas. This positional effect was most pronounced in the single pore diameter (p=0.0018) and the total pore volume ratio (p=0.0490), while other structural parameters, such as total cell count (p=0.2902), single cell diameter (p=0.2641) and total pore count (p=0.6097), remained consistent across different positions. These findings corroborate the positional effects discussed in Section 3.1.1. Integration of macroporosity and microstructural analysis revealed significant positive correlations between macroporosity (339 μm scale) and microstructural parameters (r=0.61–0.74, p<0.05), indicating that tissue-level porosity distribution is closely associated with cellular-scale architectural variation.

### 3.2. Preharvest Environmental Factors Analysis

**Figure 7:**
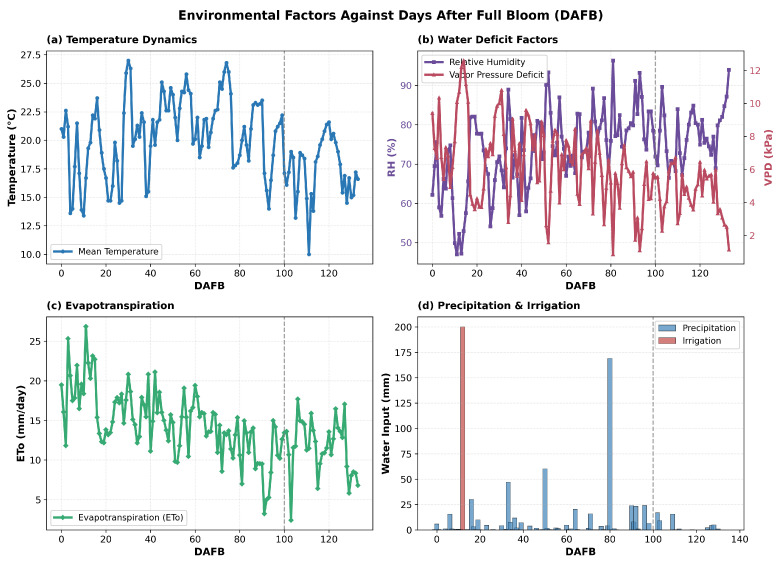
Environmental factors against DAFB.

**Figure 8:**
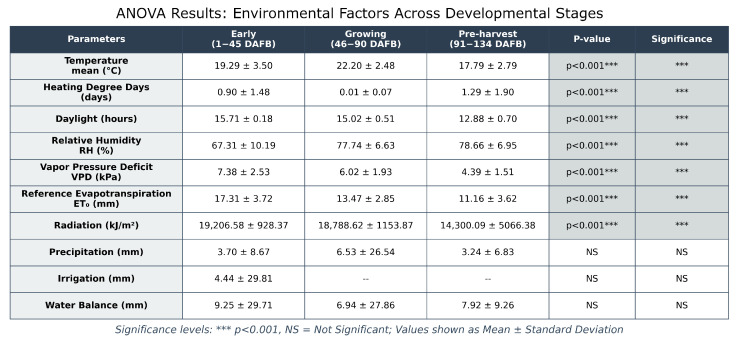
Environmental factors ANOVA difference.

Environmental monitoring throughout apple fruit development revealed distinct patterns across multiple abiotic factors (Figure 7) over the Days After Full Bloom (DAFB) period. Statistical analysis using ANOVA (Figure 8) identified three distinct developmental phases: the early season (cell division, 1–45 DAFB), the growing season (cell elongation, 46–90 DAFB), and pre-harvest maturation (91–134 DAFB). Significant differences (*p* < 0.001) were observed across these phases for several key parameters. The mean temperature decreased significantly from the growing phase (22.20 ± 2.48 °C) to the pre-harvest phase (17.79 ± 2.79 °C). The Vapor Pressure Deficit (VPD) showed a progressive decline from early season (7.38 ± 2.53 kPa) to pre-harvest (4.39 ± 1.51 kPa), while relative humidity demonstrated an inverse trend, increasing from 67.31 ± 10.19% to 78.66 ± 6.95%. Evapotranspiration (ETo) rates similarly decreased from 17.31 ± 3.72 mm/day in the early season to 11.16 ± 3.62 mm/day during pre-harvest. Solar radiation exhibited a consistent declining pattern across phases, from 19,206.58 ± 928.37 to 14,300.09 ± 5066.38 units. In particular, precipitation, irrigation, and water balance did not show significant differences between phases (NS), suggesting relatively stable water availability throughout the growing season. It can be noted that irrigation has only one value. This is because it happened only once in the early stages, therefore there is no data in the mid and late stages.

### 3.3. Preharvest Apple Growth and Drop Dynamics

**Figure 9:**
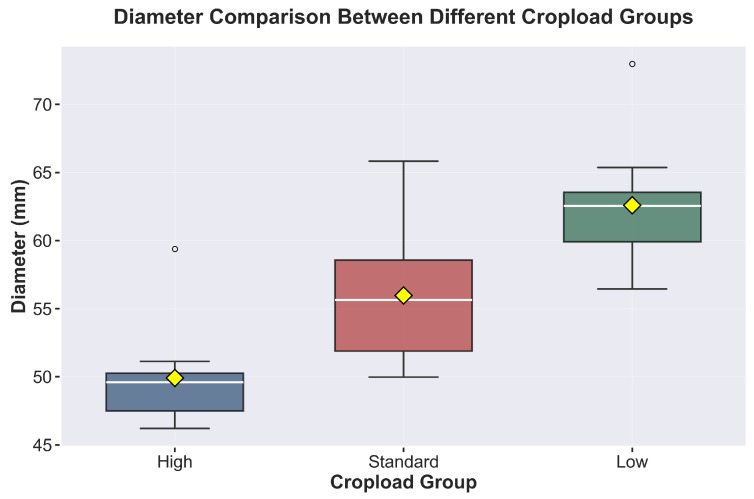
Cropload across treatments.

**Figure 10:**
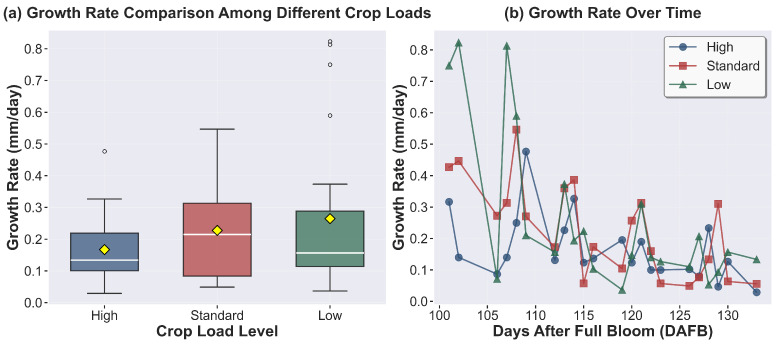
Grwoth rate across treatments.

**Figure 11:**
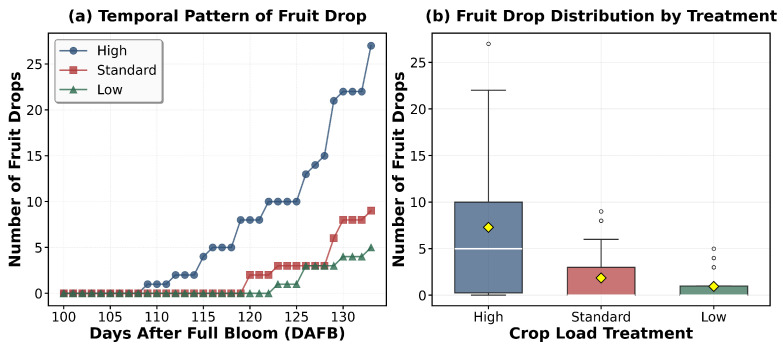
Pre-harvest fruit drop difference: (**a**) Temporal analysis; (**b**) ANOVA analysis.

Analysis of fruit development revealed significant variations across crop load treatments. Fruit diameter (Figure 9) measurements demonstrated substantial differences (*p* < 0.0001), with the low-crop fruits achieving the largest diameter (62.3 ± 2.8 mm), followed by standard (55.6 ± 3.2 mm) and high crop treatments (49.2 ± 2.1 mm). During the pre-harvest period (>100 DAFB), while growth rates did not show statistically significant differences among canopy positions or crop load treatments (*p* > 0.05), all treatments exhibited a uniform decline trend from 0.6–0.7 mm/day at 100 DAFB to 0.1–0.2 mm/day after 115 DAFB (Figure 10). Pre-harvest fruit drop (PFD) assessment (Figure 11a) during the maturation phase (100–134 DAFB) revealed significant treatment effects (Figure 11b) (*p* < 0.0001), In terms of temporal dynamics, high crop load treatments exhibited preharvest fruit drop (PFD) around 110 days after full bloom (DAFB), while standard treatments began around 120 DAFB, with low crop load treatments showing PFD at a later stage. Concerning PFD severity, with high crop load trees exhibited the highest PFD rate (29.35%) compared to standard (19.57%) and low crop load treatments (21.74%), although the drop patterns ultimately converged across all treatments after 115 DAFB.

### 3.4. Analysis of Apple Quality Parameters

**Figure 12:**
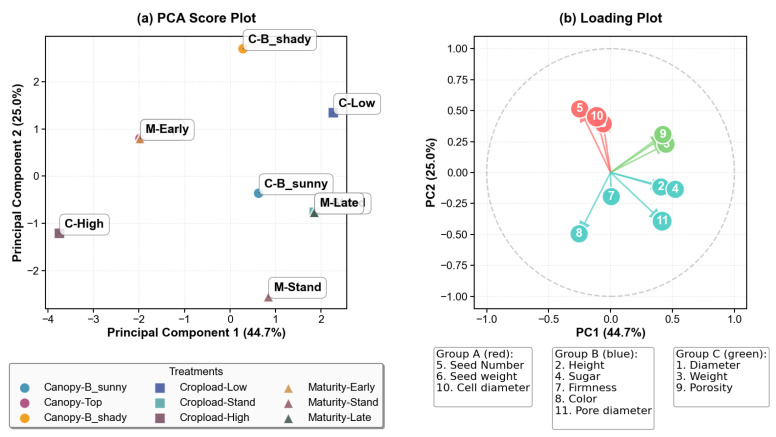
Apple quality PCA analysis.

Principal Component Analysis (Figure 12) of apple quality parameters identified three distinct groups of variables, with the first two principal components accounting for 69.7% of the total variance (PC1: 44.7%, PC2: 25.0%). Group A (seed characteristics and cell diameter) showed strong positive loadings in PC1, indicating that these traits are primarily influenced by factors associated with this component. Group B (height, sugar, firmness, color and pore diameter) exhibited moderate loadings on both axes, reflecting their combined variation, while Group C (diameter, weight, porosity) correlated strongly with PC2. The score plot visualizes how different treatments separate along these components: crop load treatments mainly differentiate along PC1 (C-High: −4.0, C-Low: 0.5) and maturity stages along PC2 (M-Stand: −2.5, M-Early: 1.0).

**Figure 13:**
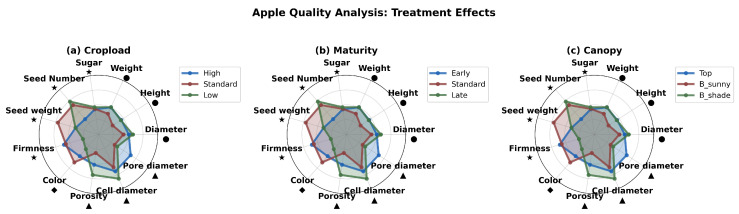
Anatomy-based Radar Charts showing fruit trait values under different conditions: (**a**) Canopy Position effect. (**b**) Cropload effect. (**c**) Maturity effect.

This indicates that internal seed traits and cell diameter are more affected by crop load, while morphological and porosity traits respond more to maturity stage. These groups represent distinct quality parameters, each responding uniquely to management practices. Group A reflects the internal structure and seed traits, Group B encompasses sensory and aesthetic attributes, and Group C pertains to morphological and internal porosity characteristics. Anatomy-based radar chart (Figure 13) complements this by illustrating treatment-specific patterns across key parameters. For example, fruits in the top canopy position and sunny exposure show improvements in Group B traits (sugar and firmness), while low crop load treatments enhance Group C traits (diameter and porosity). The early harvest fruits excel in Group B attributes, especially firmness, whereas late harvest fruits display better Group C traits, such as porosity and diameter. These visualizations collectively help identify which management practices influence specific quality parameters, providing insights on how to optimize apple quality through targeted strategies.

### 3.5. Dynamic Analysis of Environmental Factors and Apple Quality

#### 3.5.1. Single Correlation Analysis

**Figure 14:**
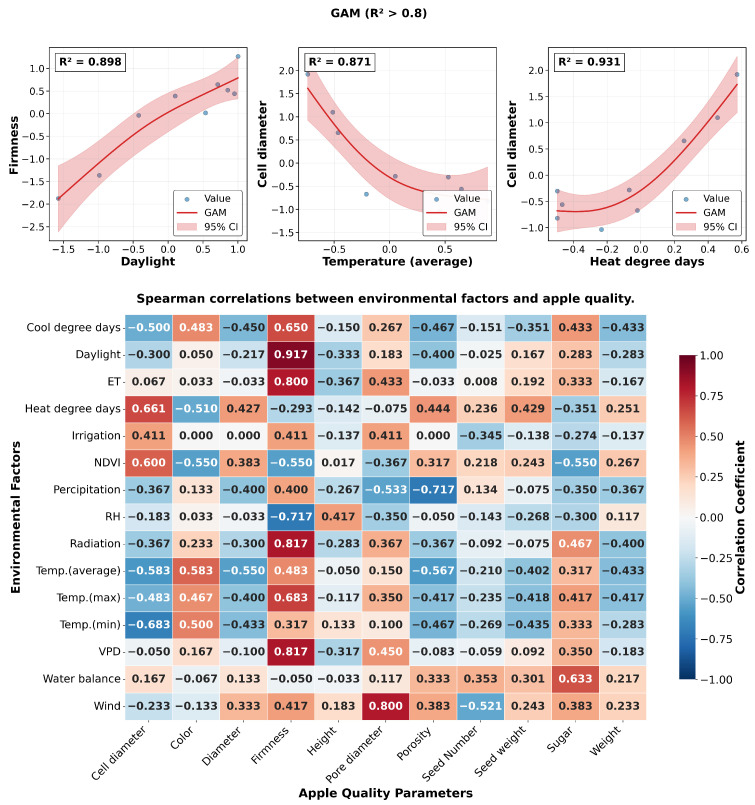
Statistical analysis: (**top**) GAM model results; (**bottom**) Spearman correlation heatmap.

The single correlation between preharvest environmental factors and apple quality parameters was investigated through both GAM (Figure 14) and Spearman correlation analysis. GAM analysis ($R^{2}>0.8$) revealed three significant nonlinear relationships: daylight showed a positive linear correlation with firmness (R^2^ = 0.898), average temperature (Taver) demonstrated a negative curvilinear relationship with cell diameter (R^2^ = 0.871), and heat degree days exhibited a U-shaped relationship with cell diameter (R^2^ = 0.931). These nonlinear patterns suggest complex physiological responses to environmental signals during fruit development.

The comprehensive Spearman correlation heatmap further validated these findings, while revealing additional significant correlations. Notably, daylight (r = 0.917, *p* < 0.001), radiation (r = 0.817, *p* < 0.007), and vapor pressure deficit (VPD) (r = 0.817, *p* < 0.007) showed strong positive correlations with fruit firmness. In contrast, relative humidity (RH) demonstrated a significant negative correlation with firmness (r = −0.717, *p* < 0.03). The observed consistency between the GAM and Spearman analyses, especially in highlighting daylight as a significant factor influencing fruit firmness, underscores the reliability of these environmental variables in affecting apple quality.

#### 3.5.2. Multi Correlations Analysis

**Figure 15:**
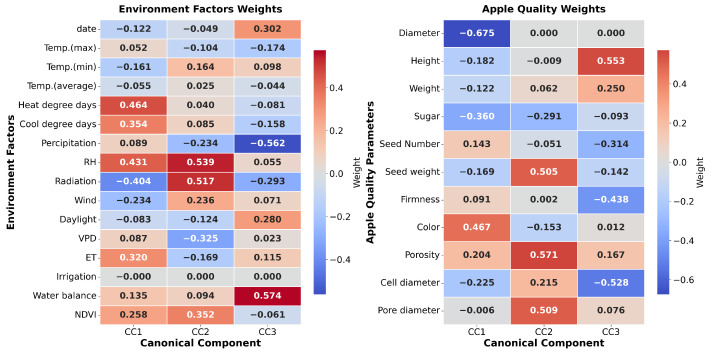
Canonical Correlation Analysis of apple quality parameters.

**Figure 16:**
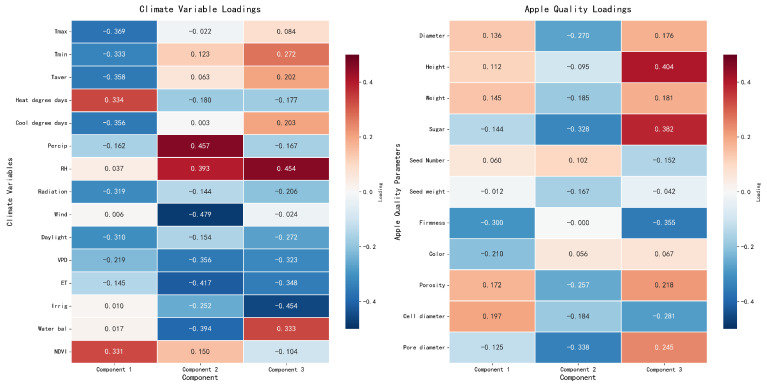
PLS analysis.

**Figure 17:**
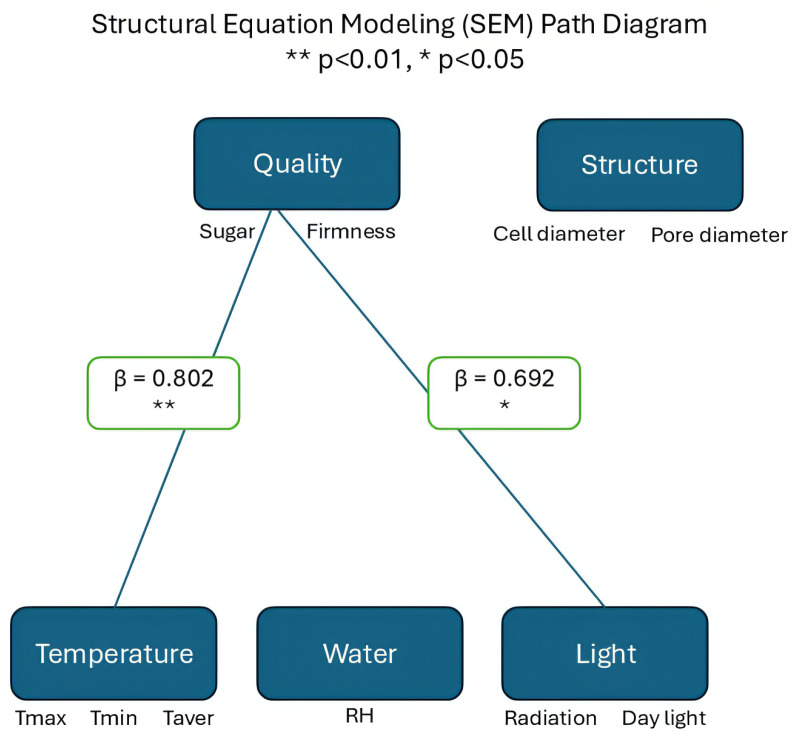
SEM path diagram.

Multidimensional statistical analyses revealed the hierarchical influence of environmental factors on apple quality parameters. Canonical Correlation Analysis (Figure 15) demonstrated three distinct component patterns, with CC1 capturing temperature-related effects (Tmax: −0.369, Tmin: −0.333), CC2 dominated by water-related variables (precipitation: 0.655, RH: 0.430) and CC3 reflecting significant fruit weight variations (−0.801). These patterns were reinforced by PLS analysis (Figure 16), which identified exceptionally high predictability for key quality indicators (sugar: R^2^ = 0.827, firmness: R^2^ = 0.903) and revealed the predominant influence of water-related variables through VIP scores (precipitation: 0.621, water balance: 0.569). The integration of these relationships through SEM (Figure 17) established a hierarchical framework in which temperature emerged as the primary driver of both fruit quality and internal structure (*p* < 0.05), while the water balance and the light environment exhibited significant indirect effects through internal structure mediation, collectively explaining the complex environmental regulation of apple development.

### 3.6. Predictive Modeling

#### 3.6.1. Apple Quality Prediction

Machine learning approaches were employed to predict apple quality based on environmental parameters, with three algorithms demonstrating varying degrees of predictive accuracy. The MLR model demonstrated the highest accuracy with R^2^ = 0.999, RMSE = 0.003, and MAE = 0.003, outperforming both Random Forest (R^2^ = 0.991, RMSE = 0.011, MAE = 0.010) and XGBoost (R^2^ = 0.944, RMSE = 0.040, MAE = 0.040) models. The best-performing MLR model utilized four environmental features, with feature importance scores revealing Taver (0.767) and Tmax (0.745) as the strongest predictors, followed by NDVI (0.187) and ET (0.170).

#### 3.6.2. Temporal Quality Prediction

The temporal analysis by predictive performance of machine learning models was evaluated across three key developmental stages (DAFB). Among all stages, the highest prediction accuracy was achieved during the cell division phase (45 DAFB) using the Random Forest model (R^2^ = 0.8925, RMSE = 0.0833), with relative humidity (0.2788), vapor pressure deficit (0.2790), and radiation-related variables (0.1396) identified as key predictors. Focusing on the critical preharvest period (134 DAFB), the XGBoost model demonstrated superior performance (R^2^ = 0.7696, RMSE = 0.1736), with wind (0.7682) and daylight (0.1455) emerging as the dominant predictive features.

## 4. Discussion

### 4.1. Innovative Application of X-Ray CT in Apple Quality Evaluation

The application of X-ray CT to assess apple tissue architecture is a novel approach in fruit quality evaluation, traditionally dominated by post-harvest and destructive methods. In this study, high-resolution X-ray CT imaging was used to analyze internal tissue structures and porosity distribution, revealing spatial heterogeneity across different tissue regions [7,8]. Three-dimensional analysis indicated that crop load significantly influenced tissue porosity, with high crop load fruits exhibiting the lowest porosity (24.7%), while late-maturity fruits showed increased porosity (28.6%) compared to early harvest ones (25%) [7]. Spatial assessments also demonstrated that tissue porosity varied with canopy position, being higher in sun-exposed areas (around 28%) than in shaded regions (approximately 26%) [8,17]. PCA effectively classified key quality traits into distinct groups, such as seed and cell diameter versus morphological and porosity attributes, accounting for 69.7% of total variance [36]. In addition, radar charts provided intuitive multidimensional visualizations of how different management practices affected parameters such as sugar content, firmness, diameter, and porosity, revealing, for example, that fruits from the upper canopy and late harvest stages scored higher in certain quality attributes [18,21]. The integration of X-ray CT imaging with multivariate statistical analysis underscores its potential as a robust, nondestructive tool for detailed internal quality assessment, offering significant advantages over traditional methods and opening new avenues for rapid, precise, and comprehensive apple quality diagnostics for both research and orchard management [5,18].

### 4.2. Evaluation of Environmental Factors and Their Correlation with Apple Quality

Multiple analytical approaches were employed to elucidate the relationships between environmental factors and apple quality parameters throughout the growing season. Initial univariate analyses using ANOVA identified distinct developmental phases—early, growing, and pre-harvest—each characterized by significant variations in abiotic conditions such as temperature, vapor pressure deficit (VPD), relative humidity (RH), solar radiation, and evapotranspiration rates (p<0.001) [6,10].

Although the meteorological station was relatively close to the experimental orchard (approximately 3 km), local microclimatic conditions within the orchard may have differed from those recorded at the station. Orchard-specific factors such as canopy structure, tree density, ground cover, soil texture and moisture, slope, and exposure can modify air temperature, relative humidity, vapor pressure deficit, radiation interception, and wind speed at the fruit and canopy level. As a result, the station-based climate data may not fully capture short-term or fine-scale microclimatic variability experienced by the fruit, potentially introducing uncertainty in the estimated strength and timing of relationships between environmental variables and fruit growth or quality responses. Despite these limitations, the meteorological station and the experimental orchard are located within the same agro-environmental context and share comparable environmental and pedological conditions, including similar soil type, topography, and land use. Therefore, the station data provide a consistent and standardized representation of the seasonal climatic trends relevant at the orchard scale, making them suitable for comparative and modeling purposes.

Notably, the mean temperature decreased progressively from the growing to pre-harvest phases, while RH increased, indicating a shift in atmospheric conditions that could influence fruit development [4,10]. Correlation analyses, including GAM and Spearman’s rank tests, revealed strong nonlinear relationships between environmental variables and fruit quality traits. For example, daylight exposure showed a positive linear correlation with firmness ($R^{2}>0.89$) [4], while the average temperature exhibited a negative curvilinear association with cell diameter ($R^{2}>0.87$) [2]. Similarly, VPD demonstrated significant positive correlations with firmness, while RH was negatively correlated [5,27]. To capture the complex hierarchical influence of environmental factors, multivariate techniques such as CCA and PLS regression were applied. CCA distinguished temperature-driven (CC1), water-related (CC2), and fruit weight-related (CC3) components, with water variables such as precipitation and RH showing prominent loadings [11]. PLS analysis further identified water balance and precipitation as key predictors of quality metrics such as sugar content and firmness, with VIP scores exceeding 0.56 [39]. SEM integrated these findings, highlighting temperature as the primary driver affecting both internal fruit structure and overall quality, with water availability and light conditions exerting significant indirect effects mediated through internal tissue properties [40,41]. Collectively, these diverse analytical methods underscore the multifaceted influence of environmental factors on apple development, emphasizing the importance of considering nonlinear and hierarchical relationships to better understand and predict fruit quality outcomes under variable environmental conditions [5,41].

#### Modeling and Key Drivers of Apple Quality Prediction

To elucidate the primary factors that influence apple quality, three machine learning algorithms—MLR, Random Forest (RF), and XGBoost—were employed to develop predictive models based on environmental data in different developmental stages. The MLR model achieved near-perfect prediction accuracy with R^2^ = 0.999, RMSE = 0.003, and MAE = 0.003, indicating a highly reliable relationship between environmental variables and quality parameters [5,42]. Feature importance analysis revealed that mean temperature (Taver) and maximum temperature (Tmax) were the most significant predictors, with importance scores of 0.767 and 0.745, respectively, underscoring the critical role of temperature in determining apple quality [10,21]. It is important to note that these represent *baseline results* from a single growing season; to mitigate overfitting, we employed training-testing dataset separation and hyperparameter tuning, though these predictions require validation across multiple seasons and cultivars before operational implementation.

Complementary analyses examined the temporal dynamics of prediction performance in key growth stages. During the early cell division phase (around 45 DAFB), the RF model demonstrated the highest accuracy (R^2^ = 0.8925), with relative humidity and vapor pressure deficit emerging as the most influential predictors [3,27]. In contrast, in the preharvest stage (134 DAFB), the XGBoost model outperformed others (R^2^ = 0.7696), with wind speed and daylight being the dominant features [43]. The dominance of wind at preharvest stages (134 DAFB) may reflect late-developmental water status regulation and cellular differentiation in response to mechanical stimuli. Wind influence likely operates through multiple pathways: enhanced transpiration affecting water balance and cell turgor, increased mechanical stress promoting cellular adjustment in cortical tissue, and altered light distribution through canopy movement affecting late-stage ripening physiology (see also Section 4.3.1). However, the exact physiological mechanism requires further investigation through controlled wind manipulation studies. These findings corroborate the importance of early stage environmental conditions, particularly temperature, and the preharvest period in shaping apple quality outcomes. In general, the results highlight temperature as the most critical environmental driver and emphasize that the early developmental phase (around 45 DAFB) is a critical window for quality prediction, supported by empirical model performance and feature importance evidence [8,11].

### 4.3. Other Effects and Considerations

#### 4.3.1. Wind Effects

Wind speed increases the thickness of the atmospheric boundary layer around leaves and fruit, enhancing transpiration rates and whole-tree evapotranspiration, particularly under conditions of moderate to high vapor pressure deficit. Over the season, sustained wind exposure can therefore lead to repeated or prolonged periods of mild water stress, even when soil water availability is not limiting. Such conditions reduce xylem inflow to the fruit more strongly than phloem inflow, altering the balance between water and carbon accumulation in developing tissues. At late developmental stages (around 134 DAFB), when xylem functionality in apple fruit is already reduced and growth is mainly sustained by phloem import, wind-induced increases in transpiration can further limit fruit water uptake. This favors higher dry matter concentration, reduced cell turgor during expansion, and progressive enlargement of intercellular spaces rather than uniform cell enlargement. As documented in eco-physiological studies of apple fruit growth, these processes contribute to changes in tissue density and porosity, particularly during the final stages of maturation. Therefore, the emergence of wind as a dominant predictor in the XGBoost model at 134 DAFB likely reflects its role as an integrative proxy for cumulative evaporative demand rather than a direct mechanical effect. Wind speed encapsulates seasonal-scale modulation of transpiration, water fluxes, and fruit hydration status, which are known to influence cell separation, intercellular space development, and overall fruit microstructure during late maturation stages.

#### 4.3.2. Rootstock Effects

Rootstocks are known to influence tree vigor, canopy architecture, water relations, nutrient uptake, and carbon allocation, all of which indirectly affect fruit growth dynamics and fruit sensitivity to environmental conditions such as temperature, water stress, and light availability. Differences in root hydraulic conductivity, root-to-shoot signaling (including hormonal balance), and nutrient absorption efficiency among rootstocks can alter the tree’s capacity to buffer abiotic stress, thereby modifying fruit growth rates, cell division, and cell expansion processes. As highlighted in the literature on pre-harvest apple development and precision orchard management, rootstock-mediated effects on water status and carbon availability are particularly critical during early developmental stages, when fruit growth is most sensitive to environmental constraints. Moreover, rootstocks can influence the timing and intensity of physiological responses to stress by affecting xylem functionality, transpiration rates, and stomatal regulation, which in turn interact with temperature and vapor pressure deficit to shape fruit development. Consequently, part of the variability observed in fruit responses to abiotic drivers may reflect not only environmental conditions but also genotype-dependent rootstock effects.

#### 4.3.3. Practical Applications and Implementation Considerations

Despite the costly equipment used for the study purpose, one of the objective of the study is to identify environmental key factors that can easly sensed, to provide tools for the farmers that can practically be used with already existing installations. Growers can realistically benefit from these findings in several ways:

1. Targeted management timing—The identification of early developmental stages as critical for final fruit quality supports existing low-cost practices such as optimized thinning timing, irrigation scheduling, and canopy management, which are already widely accessible to growers.
2. Simplified sensor adoption—While CT imaging itself is not farm-ready, the environmental drivers identified (temperature, light interception, water status) can be monitored using relatively inexpensive weather stations, soil moisture sensors, and proximal or remote sensing tools that are increasingly common in commercial orchards.
3. Decision-support systems (DSS)—The relationships quantified in this study contribute to the development of predictive models and DSS platforms that translate complex physiological responses into actionable recommendations (e.g., irrigation thresholds, crop load adjustment), without requiring growers to interact with the underlying complex technology.
4. Long-term orchard design and strategy—Insights into the roles of crop load, canopy position, light distribution, and temperature can inform structural decisions such as training systems, row orientation, and planting density, which are one-time or infrequent investments rather than recurring high-tech costs.

### 4.4. Limitations

Although this study presents comprehensive information on apple fruit quality development, several limitations should be acknowledged. The analysis was conducted on a limited sample size (n = 27 apples for low-resolution scanning, n = 9 for high-resolution microstructural analysis) from a single growing season, which can restrict generalizability across cultivars and environments. Although we implemented training-testing dataset separation and hyperparameter tuning to mitigate overfitting risks, these baseline findings require validation using independent datasets from multiple seasons and diverse cultivars with cross-validation approaches to enhance robustness and reliability of predictive models. Additionally, the linear regression calibration for grey value-porosity conversion was established using a preliminary 10-pixel validation sample specific to ‘Gala’ apples. While this approach follows established protocols from prior X-ray CT studies and the analyzed scans displayed a relatively homogeneous pattern, this limited sampling size may not fully capture the inherent spatial heterogeneity of apple parenchyma, which is characterized by marked variability in cell size, intercellular spaces, and tissue organization across cortex and core regions. Therefore, to confirm the universality and robustness of the grey value-porosity relationship, future validation should utilize a substantially larger number of voxels distributed across multiple anatomical zones and diverse cultivars. All experimental trees were grafted on M106 rootstock, which standardized the rootstock effect but may limit model generalization. Different rootstocks (e.g., M7, MM106, Geneva series) exhibit varying hydraulic properties, nutrient uptake characteristics, and stress tolerance, which could modulate the environmental-quality relationships identified in this study. Multi-rootstock validation across different rootstock-cultivar combinations is recommended to assess whether the identified relationships are consistent across rootstock types or show rootstock-dependent variation in environmental responsiveness. Furthermore, the observed relationships between climatic data and fruit quality traits should be interpreted with caution, as fine-scale microclimatic variability within the orchard canopy may not be fully captured, as the information was primarily gathered from the available weather station not exactly located within the trial orchard. Future studies would benefit from deploying on-site microclimate sensors within the canopy to further reduce uncertainty and improve the resolution of environment–fruit interactions.

### 4.5. Future Directions

Building on the insights gained from this work regarding environment-quality relationships, future research should prioritize multi-season validation across diverse cultivars and climates to confirm the robustness of these baseline models. Development of real-time predictive models integrating environmental monitoring data will enable dynamic orchard management decisions, supporting more precise and adaptive practices [5,34]. Further work on faster and more cost-effective imaging techniques, combined with operational validation in diverse growing environments, will facilitate commercial application and improve resilience to climate variability, ultimately advancing sustainable and precision fruit production [7,41].

## 5. Conclusions

This study presents a novel and comprehensive framework that integrates dual-resolution X-ray computed tomography (X-ray CT), environmental data, and advanced analytical techniques, including machine learning and innovative visualization methods, to elucidate the mechanisms underlying apple fruit quality development. Our findings underscore temperature as the predominant environmental regulator, particularly during the early developmental stages, with multiple analytical approaches, such as correlation analysis, canonical correlation analysis, and machine learning models, consistently confirming its critical role. The application of X-ray CT enabled unprecedented insights into internal cellular architecture, with structural parameters demonstrating high predictive power for key quality attributes ($R^{2}>0.89$), highlighting the innovative use of this technology in apple quality evaluation.

## Data Availability

The raw data supporting the conclusions of this article will be made available by the authors on request.

## Abbreviations

The following abbreviations are used in this manuscript:

CT	Computed Tomography
PCA	Principal Component Analysis
CCA	Canonical Correlation Analysis
SEM	Structural Equation Modeling
DABF	Days After Full Bloom
ML	Machine Learning
MLR	Multiple Linear Regression
RF	Random Forest
ET	Evapotransipration
VPD	Vapor Pressure Deficit
DICOM	Digital Imaging and Communications in Medicine
ANOVA	Analysis of Variance
GAM	Generalized Additive Models
PLS	Partial Least Squares
RMSE	Root Mean Square Error
MAE	Mean Absolute Error
PFD	Preharvest Fruit Drop
RH	Relative Humidity
VIP	Variable Importance in Projection

## References

[1] Arseneault M.H., Cline J.A. (2016). A review of apple preharvest fruit drop and practices for horticultural management. Sci. Hortic..

[2] Warrington I.J., Fulton T.A., Halligan E.A., de Silva H.N. (1999). Apple fruit growth and maturity are affected by early season temperatures. J. Am. Soc. Hortic. Sci..

[3] Zude-Sasse M., Pérez-Cutillas P., Akca A., Theuvsen L., Tsoulias N. (2022). Effects of soil ECa and LiDAR-derived leaf area on yield and fruit quality in apple production. Biosyst. Eng..

[4] Ahmadi-Afzadi M., Tahir I., Nybom H. (2013). Impact of harvesting time and fruit firmness on the tolerance to fungal storage diseases in an apple germplasm collection. Postharvest Biol. Technol..

[5] Akter T., Kim W.S., Lee Y. (2024). A comprehensive review of external quality measurements of fruits and vegetables using nondestructive sensing technologies. J. Agric. Food Res..

[6] Herold B., Truppel I., Zude M., Geyer M. (2005). Spectral measurements on ‘Elstar’ apples during fruit development on the tree. Biosyst. Eng..

[7] Defraeye T., Verboven P., Nicolaï B.M., Ho Q.T. (2021). X-ray CT and porosity mapping to determine the effect of ‘Fuji’ apple morphological and microstructural properties on the incidence of CO_2_-induced internal browning. Sci. Rep..

[8] Verboven P., Nicolaï B.M., Defraeye T. (2021). X-ray computed tomography for quality inspection of agricultural products: A review. Comput. Electron. Agric..

[9] Huynh H.X., Lam B.H., Le H.V.C., Le T.T., Duong-Trung N. (2024). Design of an IoT ultrasonic-vision based system for automatic fruit sorting utilizing size and color. Internet Things.

[10] Minas I.S., Tanou G., Molassiotis A. (2018). Environmental and orchard bases of peach fruit quality. Sci. Hortic..

[11] Auzmendi I., Mata M., Lopez G., Girona J., Marsal J. (2011). Intercepted radiation by apple canopy can be used as a basis for irrigation scheduling. Agric. Water Manag..

[12] Anthony B.M., Minas I.S. (2022). Redefining the impact of preharvest factors on peach fruit quality development and metabolism: A review. Sci. Hortic..

[13] Argenta L.C., Wood R.M., Mattheis J.P., Thewes F.R., Nesi C.N. (2024). Impact of storage atmosphere relative humidity on ’Gala’ apple fruit quality. Postharvest Biol. Technol..

[14] Li W., Liu Z., Wang H., Zheng Y., Zhou Q. (2024). Harvest maturity stage affects watercore dissipation and postharvest quality deterioration of watercore ‘Fuji’ apples. Postharvest Biol. Technol..

[15] Li S., Wang P., Zhang H., Li M., Wang X. (2021). Biogenic volatile organic compound emissions from leaves and fruits of apple and peach trees during fruit development. J. Environ. Sci..

[16] Manfrini L., Bonora A. (2024). Advances in Understanding Pre-Harvest Apple Fruit Development.

[17] Nicolaï B.M., Verboven P., Piazza L., Datta A.K. (2021). X-ray computed tomography for quality inspection of agricultural produce. Annu. Rev. Food Sci. Technol..

[18] Magwaza D., Opara U.L., Cronje P.J., Landahl S., Nieuwoudt H.H., Mouazen A.M., Terry L.A. (2020). Combination of shape and X-ray inspection for apple internal quality control: In silico analysis of the methodology based on X-ray computed tomography. J. Food Eng..

[19] Huang M., Zhang M., Wang Q., Zhu Q. (2021). Inline nondestructive internal disorder detection in pear fruit using explainable deep anomaly detection on X-ray images. Comput. Electron. Agric..

[20] Nicolaï B.M., Verboven P., Defraeye T., De Ketelaere B., Rogge S., Ho Q.T., Hertog M.L. (2020). Unsupervised anomaly detection for pome fruit quality inspection using X-ray radiography. Comput. Electron. Agric..

[21] Verboven P., Defraeye T., Nicolaï B.M. (2018). Assessment of bruise volumes in apples using X-ray computed tomography. Food Control.

[22] Verboven P., Defraeye T., Nicolaï B.M. (2020). Oxygen diffusivity mapping of fruit and vegetables based on X-ray CT. Food Bioprocess Technol..

[23] Van de Looverbosch M., Verboven P., Nicolaï B.M., Defraeye T. (2021). Computed tomography imaging-based bitter pit evaluation in apples. Comput. Electron. Agric..

[24] Wood R.M., Schut D.E., Trull A.K., Marcelis L.F.M., Schouten R.E. (2024). Detecting internal browning in apple tissue as determined by a single CT slice in intact fruit. Postharvest Biol. Technol..

[25] Mirbod O., Choi D., Heinemann P.H., Marini R.P., He L. (2023). On-tree apple fruit size estimation using stereo vision with deep learning-based occlusion handling. Biosyst. Eng..

[26] Matsui T., Kamata T., Koseki S., Koyama K. (2022). Development of automatic detection model for stem-end rots of ‘Hass’ avocado fruit using X-ray imaging and image processing. Postharvest Biol. Technol..

[27] Iglesias I., Salvia J., Torguet L., Cabús C. (2002). Orchard cooling with overtree microsprinkler irrigation to improve fruit colour and quality of ‘Topred Delicious’ apples. Sci. Hortic..

[28] Wang B., Yang X., Chen J. (2024). A smart fruit size measuring method and system in natural environment. J. Food Eng..

[29] Beckles D.M. (2021). Potential link between fruit yield, quality parameters and phytohormonal changes in preharvest UV-C treated strawberry. Sci. Hortic..

[30] Miranda J.C., Rosell-Polo J.R., Escolà A., Arnó J., Martínez-Casasnovas J.A. (2023). Assessing automatic data processing algorithms for RGB-D cameras to predict fruit size and weight in apples. Comput. Electron. Agric..

[31] Gené-Mola J., Sanz-Cortiella R., Rosell-Polo J.R., Morros J.R., Ruiz-Hidalgo J., Vilaplana V. (2023). Looking behind occlusions: A study on amodal segmentation for robust on-tree apple fruit size estimation. Comput. Electron. Agric..

[32] Saha K.K., Montero J.I., Muñoz P., Baeza E.J. (2024). Chlorophyll content estimation and ripeness detection in tomato fruit based on NDVI from dual wavelength LiDAR point cloud data. J. Food Eng..

[33] Huang M., Wang Q., Zhang M., Zhu Q. (2020). BraeNet: Internal disorder detection in ‘Braeburn’ apple using X-ray imaging data. Comput. Electron. Agric..

[34] Herremans E., Verboven P., Bongaers E., Estrade P., Verlinden B.E., Wevers M., Hertog M.L., Nicolaï B.M. (2013). Characterisation of ‘Braeburn’ browning disorder by means of X-ray micro-CT. Postharvest Biol. Technol..

[35] Qu J., Zhang M., Zhu Q., Wang Q. (2020). Nondestructive internal disorders detection of ‘Braeburn’ apple fruit by X-ray dark-field imaging and machine learning. Food Control.

[36] Nicolaï B.M., Defraeye T., De Ketelaere B., Herremans E., Hertog M.L., Saeys W., Verboven P. (2019). Assessment of apple bruise resistance under transient collisions through X-ray computed tomography and image processing. Postharvest Biol. Technol..

[37] Mebatsion H.K., Verboven P., Endalew A.M., Billen J., Ho Q.T., Nicolaï B.M. (2009). A novel method for 3-D microstructure modeling of pome fruit tissue using synchrotron radiation tomography images. J. Food Eng..

[38] Wang J., Liu H., Zhang H. (2020). Non-destructive internal disorder segmentation in pear fruit by X-ray radiography and AI. IEEE Access.

[39] Chen L., Sun C., Cao Y., Li M. (2021). From sunburn detection to optimal cooling: A review of recent applications of thermal imaging to improve preharvest and postharvest handling of fruit and vegetables. Trends Food Sci. Technol..

[40] Defraeye T., Verboven P., Nicolaï B.M. (2019). Porosity quantification in pear fruit with X-ray CT and spatially resolved spectroscopy. Postharvest Biol. Technol..

[41] Verboven P., Nicolaï B.M., Defraeye T. (2020). Investigating non-destructive quantification and characterization of pomegranate fruit internal structure using X-ray computed tomography. J. Food Eng..

[42] Atamian H.S., Davila F.E.L., Prakash A. (2023). A transcriptomic study of ‘Granny Smith’ apple fruit response to x-ray irradiation using RNA-Seq. Sci. Hortic..

[43] Li Y., Zhang M., Wang Q., Zhu Q. (2020). Synthetic data for X-ray CT of healthy and disordered pear fruit using deep learning. Sci. Data.

